# Flubendazole, FDA-approved anthelmintic, targets breast cancer stem-like cells

**DOI:** 10.18632/oncotarget.3436

**Published:** 2015-01-21

**Authors:** Zhi-Jie Hou, Xi Luo, Wei Zhang, Fei Peng, Bai Cui, Si-Jin Wu, Fei-Meng Zheng, Jie Xu, Ling-Zhi Xu, Zi-Jie Long, Xue-Ting Wang, Guo-Hui Li, Xian-Yao Wan, Yong-Liang Yang, Quentin Liu

**Affiliations:** ^1^ Institute of Cancer Stem Cell, Dalian Medical University, Dalian, China, Sun Yat-sen University Cancer Center, State Key Laboratory of Oncology in South China, Collaborative Innovation Center of Cancer Medicine, Guangzhou, China; ^2^ Center for Molecular Medicine, School of Life Science and Biotechnology, Dalian University of Technology, Dalian, China; ^3^ Department of Hematology, Third Affiliated Hospital, Sun Yat-sen University, Guangzhou, China; ^4^ Laboratory of Molecular Modeling and Design, State Key Laboratory of Molecular Reaction Dynamics, Dalian Institute of Chemical Physics, The Chinese Academy of Sciences, Dalian, China; ^5^ Department of Critical Care Medicine, the First Affiliated Hospital, Dalian Medical University, Dalian, China

**Keywords:** flubendazole, breast cancer, cancer stem-like cell, cell cycle, tubulin

## Abstract

Cancer stem-like cell (CS-like cell) is considered to be responsible for recurrence and drug resistance events in breast cancer, which makes it a potential target for novel cancer therapeutic strategy. The FDA approved flubendazole, has been widely used in the treatment of intestinal parasites. Here, we demonstrated a novel effect of flubendazole on breast CS-like cells. Flubendazole inhibited breast cancer cells proliferation in dose- and time-dependent manner and delayed tumor growth in xenograft models by intraperitoneal injection. Importantly, flubendazole reduced CD44^high^/CD24^low^ subpopulation and suppressed the formation of mammosphere and the expression of self-renewal related genes including *c-myc, oct4, sox2, nanog* and *cyclinD1*. Moreover, we found that flubendazole induced cell differentiation and inhibited cell migration. Consistently, flubendazole reduced mesenchymal markers (β-catenin, N-cadherin and Vimentin) expression and induced epithelial and differentiation marker (Keratin 18) expression in breast cancer cells. Mechanism study revealed that flubendazole arrested cell cycle at G2/M phase and induced monopolar spindle formation through inhibiting tubulin polymerization. Furthermore, flubendazole enhanced cytotoxic activity of conventional therapeutic drugs fluorouracil and doxorubicin against breast cancer cells. In conclusion, our findings uncovered a remarkable effect of flubendazole on suppressing breast CS-like cells, indicating a novel utilization of flubendazole in breast cancer therapy.

## INTRODUCTION

Breast cancer is one of the most frequently diagnosed cancer and the leading cause of cancer death among women worldwide [1]. Despite conventional therapies (surgery, chemo-radiotherapy and hormonal therapy) have improved the survival rates in breast cancer patients [2-4], recurrence and metastasis are still inevitable. CS-like cells (also known as stemloids) attract much more attention owing to their contribution to therapy resistance, cancer metastasis and relapse in breast cancer [5-8]. CS-like cells are characterized by the expression of biomarkers such as CD44^high^/CD24^low^ and aldehyde dehydrogenase1 (ALDH1) [8, 9]. Activated signaling pathways such as Hedgehog (Hh), Wnt/β-catenin, and Notch play key roles in the regulation of self-renewal and differentiation in breast CS-like cells [10].

Breast CS-like cells are enriched by chemotherapy and radiotherapy, indicating therapy resistance of CS-like cells [6, 7]. Chemotherapy drugs including doxorubicin and cyclophosphamide increase the percentage of CD44^high^/CD24^low^ population and the formation of mammosphere in residual tumors [6]. Moreover, breast CS-like cells acquire resistance to radiation by producing low level of reactive oxygen species (ROS) and phosphorylated H2AX [7]. Activation of Notch-1 signaling pathway is considered to be responsible for radiotherapeutic resistance of CS-like cells [7].

Epithelial-mesenchymal transition (EMT) is a key process during cancer metastasis. Breast CS-like cells display EMT phenotype by loss of adhesion protein E-cadherin and increased expression of fibronectin and Vimentin, indicating that EMT is involved in the regulation of CS-like cell properties [5]. Ectopic expression of Twist or Snail induces EMT phenotype in human mammary epithelial cells [5]. Interestingly, the induction of EMT leads to CD44^high^/CD24^low^ population enrichment and mammosphere formation [5] by H-Ras activation [11]. Besides, the expression of CS-like cell markers including CD44^high^/CD24^low^, ALDH1, CD44v6 and ABCG2, contributes to tumor local recurrence and poor clinical outcome in breast cancer patients [8, 12, 13]. Therefore, strategies targeting CS-like cells provide great promise for preventing therapy resistance, metastasis, and recurrence in breast cancer.

CS-like cells are proposed to be a distinguished sub-group of proliferating cells concomitant with activating self-renewal capability [14, 15]. Thus, CS-like cells show proliferating ability different from that for cancer stem cells (CSCs). Recent studies have shown that cell cycle machinery was involved in the regulation of cancer stem-like cell capabilities [16-18]. For instance, depletion of the CS-like cell marker USP22 arrests cell cycle at G1 phase in lung cancer cells [16]. Moreover, ectopic miR-34a expression induces cell cycle blockage and inhibits clonogenic expansion and tumor development by targeting CD44 in prostate CS-like cells [17, 18]. Consistently, silence of cell cycle protein Aurora-A suppresses OCT4 and NANOG expression coupled with cell cycle blockage at G2/M phase in ESCs [19, 20]. Therefore, the modulation of cell cycle progression may open an avenue for targeting CS-like cells.

Flubendazole, a member of benzimidazole families, has the typical benzimidazole moiety but with an added fluorine atom as the major structure, which makes it different from other benzimidazoles [21]. Flubendazole is widely used as a safe and efficacious anthelmintic drug for gastrointestinal parasites control in human, rodents and ruminants [21-25]. It disrupts tubulin polymerization by showing preference for binding to nematode tubulin rather than the tubulin in host [26, 27]. Recent studies report that flubendazole has additional function in inhibiting cell growth in leukemia and intestinal cancer [28, 29]. Flubendazole suppresses cell proliferation *in vitro*, delays tumor formation in xenograft models and displays preclinical activity by inhibiting tubulin polymerization in leukemia and lymphoma [29]. Notably, vinblastine resistant cells overexpressing P-glycoprotein are sensitized to flubendazole, suggesting that flubendazole is able to overcome vinblastine resistance in leukemia and lymphoma [29].

In the present study, we demonstrated that flubendazole, a FDA-approved anthelmintic drug, inhibited cancer cell proliferation *in vitro* and *in vivo* at clinically tolerable concentration. We showed that flubendazole targeted breast CS-like cells through disrupting cell cycle progression. Moreover, flubendazole suppressed cell migration, induced cell differentiation and enhanced conventional chemotherapeutic efficiency in breast cancer cells. These new data suggested the potential utilization of flubendazole in breast cancer treatment by targeting CS-like cells.

## RESULTS

### Flubendazole inhibits cell proliferation in human breast cancer cells

**Figure 1:**
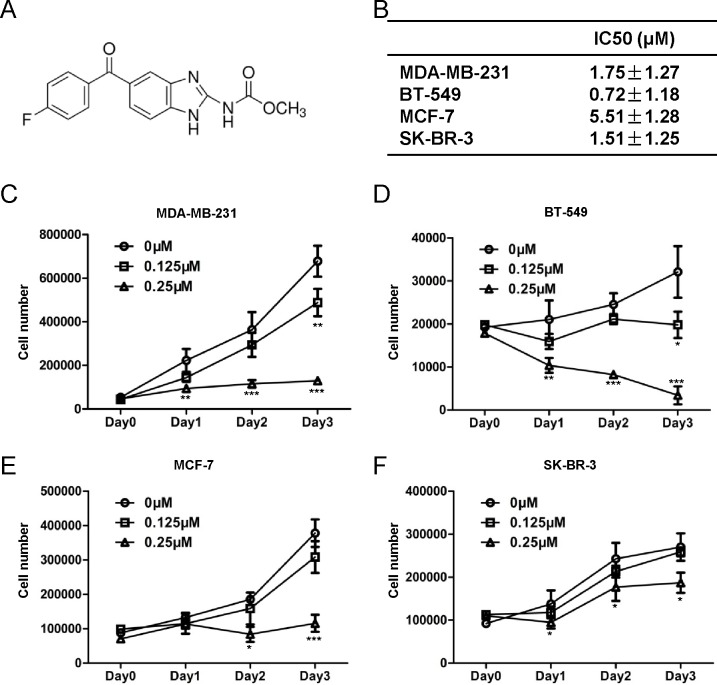
Flubendazole inhibits cell proliferation in human breast cancer cells (A) Chemical structure of flubendazole. (B) The IC50 of flubendazole measured by sigmoidal curve fitting in MDA-MB-231, BT-549, MCF-7 and SK-BR-3 cells. (C) MDA-MB-231, (D) BT-549, (E) MCF-7 and (F) SK-BR-3 cells were treated with increasing concentration of flubendazole (from 0 to 0.25 μM) respectively. After 24, 48 and 72 hr of incubation, cell proliferation was measured by cell counting assay. Data from three independent experiments were shown as mean ± S.D. (*p<0.05, **p<0.01, ***p<0.001, Student's t test) compared with vehicle-treated breast cancer cells.

The chemical structure of flubendazole was depicted in (Fig. 1A). To identify the cytotoxic effect of flubendazole in breast cancer cells, MDA-MB-231, BT-549, MCF-7 and SK-BR-3 cells were treated with increasing concentration of flubendazole (from 0 to 8μM) for 24, 48 and 72 hr, respectively. Cell viability was determined by MTT assay. Results showed that flubendazole significantly reduced cell viability in breast cancer cells (Fig. S1A-D). The 50% inhibitory concentration (IC50) measured by sigmoidal curve fitting in MDA-MB-231, BT-549, MCF-7 and SK-BR-3 cells were 1.75 ± 1.27, 0.72 ± 1.18, 5.51 ± 1.28 and 1.51 ± 1.25 μM, respectively (Fig. 1B). Moreover, the significant inhibition of cell proliferation in both dose- and time-dependent manners in MDA-MB-231, BT-549, MCF-7 and SK-BR-3 cells was confirmed by cell counting assay (Fig. 1C-F). Flubendazole inhibited cell proliferation in MDA-MB-231, MCF-7 and SK-BR-3 cells, while a severe cytotoxic effect was observed in BT-549 cells. These data indicated that flubendazole played diverse roles in breast cancer cells.

### Flubendazole delays tumor growth in xenograft model

**Figure 2:**
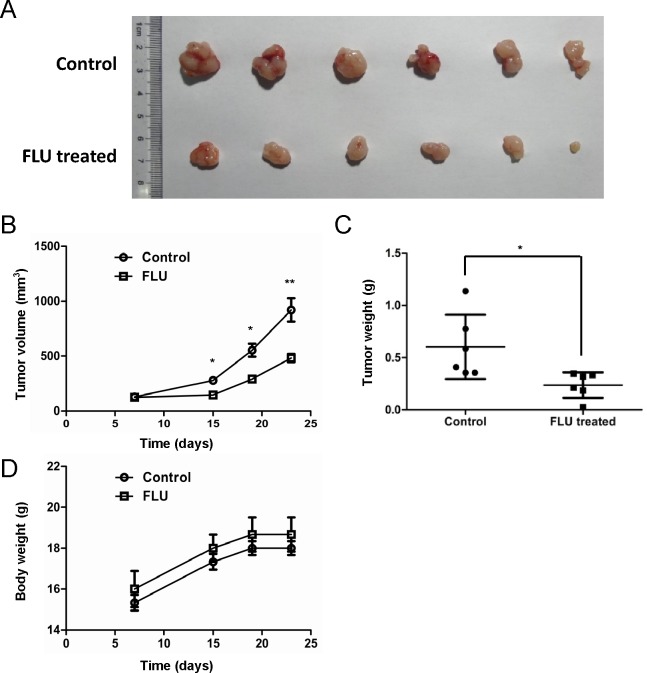
Flubendazole delays tumor growth in xenograft model (A) Tumors were generated with MDA-MB-231 breast cancer cells. MDA-MB-231 cells were inoculated into the right flank of nude mice. When the tumors developed for 7 days, mice were randomly distributed into two groups, flubendazole treated (25 mg/kg) or vehicle treated. (B) Tumor growth curves were monitored during the experimental period (n=6). (C) Tumor weight was presented by scatter plot. (D) The body weight of the mice was monitored during the experiment. Data represent the mean ± S.D. of three independent experiments (*p<0.05, **p<0.01, Student's t test).

As flubendazole displayed anti-proliferation activity on malignant breast cancer cells *in vitro*, we further evaluated whether flubendazole inhibited tumorigenicity *in vivo* by using a xenograft tumor model. We subcutaneously inoculated MDA-MB-231 cells into the right flank of nude mice. When the tumors developed for 7 days (~100 mm^3^), mice were randomized to receive flubendazole (20 mg/kg, once daily) or vehicle control intraperitoneally. After 16 days of treatment, tumors in flubendazole treated group (357.97 ± 37.3 mm^3^, *p*=0.0036) were smaller than that in control group (792.03 ± 105.83 mm^3^), (Fig. 2A-B). Consistently, tumor weight in flubendazole treated group (0.24 ± 0.12 g, *p*=0.033) was lower than that in control group (0.6 ± 0.31 g), (Fig. 2C). These findings indicated that tumor growth in xenograft model was significantly suppressed by flubendazole. Importantly, mice in flubendazole treated group showed no obvious difference in body weight compared to control group (Fig. 2D). In addition, mouse behavior, feeding pattern and overall activity did not show significant changes. These results thus indicated that flubendazole inhibited tumorigenicity efficiently in xenograft model with favorable toxicology profiles.

### Flubendazole reduces CS-like cell properties in breast cancer cells

**Figure 3:**
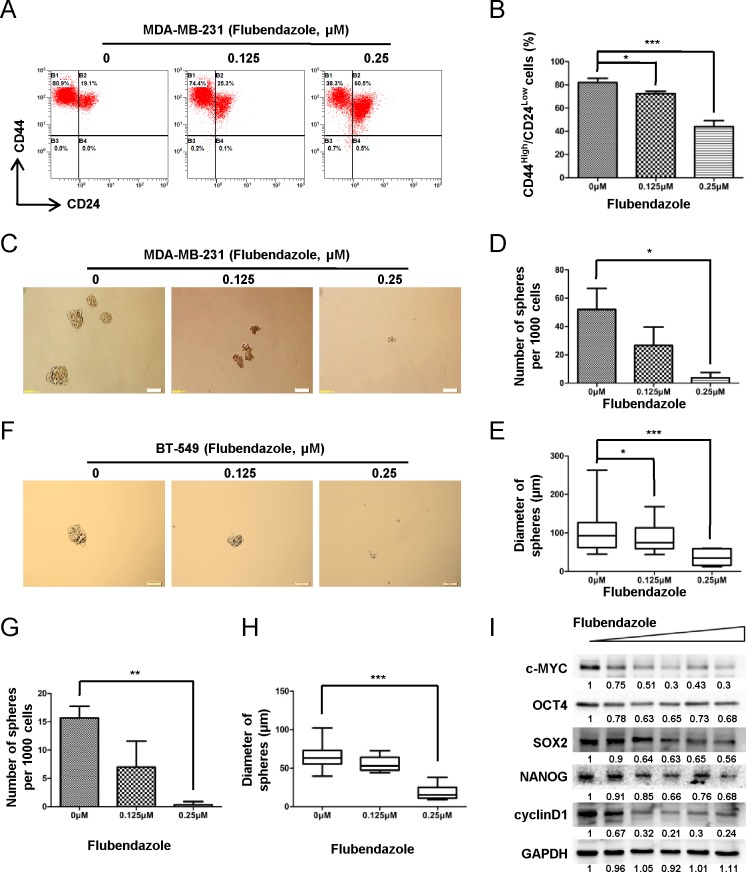
Flubendazole reduces CS-like cell properties in breast cancer cells (A) CD44^high^/CD24^low^ population was analyzed by flow cytometry analysis in MDA-MB-231 cells with or without flubendazole treatment. (B) The statistical results of three independent experiments were presented. Data were demonstrated as mean ± S.D. (*p<0.05, ***p<0.001, Student's t test). (C) MDA-MB-231 cells were plated in low-serum non-adherent culture conditions. Images were obtained by microscopy at 10× magnification and were representative of the mammosphere formed after 7 days in culture (Scale bar=100 μm). (D) The number of MDA-MB-231 sphere with > 60 μm in diameter obtained from 10^3^ cells. Data were demonstrated as mean ± S.D. of three independent experiments (*p<0.05, Student's t test). (E) The box plot graph showed the mammosphere size data from three independent experiments. Data were demonstrated as mean ± S.D. of three independent experiments (*p<0.05, ***p<0.001, Student's t test). (F) BT-549 cells were treated under the same condition as above. Representative images of BT-549 mammosphere were shown. (G-H) The number and the size of BT-549 spheres were counted. Data were presented as mean ± S.D. of three independent experiments (**p<0.01, ***p<0.001, Student's t test). (I) MDA-MB-231 cells were treated with flubendazole in increasing doses (from 0 μM to 2 μM) and subjected to western blot. The expression of c-MYC, OCT4, SOX2, NANOG and cyclinD1 was identified. GAPDH acted as the loading control. The quantification of blot density was shown in the figure.

To identify whether flubendazole inhibits breast cancer stem-like cells, we performed flow cytometry analysis to analyze the proportion of CS-like cell subpopulation in breast cancer cells based on the expression of CD44 and CD24 (CD44^high^/CD24^low^configuration). As shown in (Fig. 3A, MDA-MB-231 cells were enriched with CD44^high^/CD24^low^ fraction (~80%). After 2 days of flubendazole (0, 0.125 and 0.25 μM) treatment, CD44^high^/CD24^low^population in MDA-MB-231 cells was dramatically reduced from 81.97 ± 3.62% to 72.43 ± 1.98% and 44.07 ± 5.20% with increasing flubendazole concentration (from 0 to 0.25 μM, *p*<0.05) (Fig. 3B). This finding raised the possibility that flubendazole might inhibit CS-like cell capability in breast cancer cells.

We subsequently tested whether flubendazole inhibited anchorage-independent spheres formation. MDA-MB-231 cells were exposed to indicated doses of flubendazole (0, 0.125 and 0.25 μM) for 7 days. Representative results were shown in (Fig. 3C). Flubendazole significantly reduced the number (>60 μm) and size of mammospheres (Fig. 3D-E). Similar results were also observed in BT-549 cells (Fig. 3F-H). Furthermore, flubendazole decreased the expression of self-renewal genes such as *c-myc, oct4, sox2, nanog and cyclinD1* in MDA-MB-231 cells (Fig. 3I). Collectively, these data displayed that flubendazole dramatically reduced CS-like cell properties in breast cancer cells.

We previously demonstrated that epirubicin-resistant MCF-7 cells (epi-MCF-7) were enriched with CD44^high^/CD24^low^ population together with an increased expression of self-renewal related genes including *β-catenin*, *c-myc*, *sox2*, *oct4* and *nanog* compared with wild-type MCF-7 cells [30]. We confirmed that epi-MCF-7 had approximately 64% of CD44^high^/CD24^low^ subpopulation (Fig. S2A, right panel), while only as few as 0.1% of CD44^high^/CD24^low^ population was maintained in MCF-7 cells (Fig. S2A, left panel). MTT and cell counting assays were performed to evaluate the cytotoxic effect of flubendazole in both MCF-7 and epi-MCF-7 cells. Results showed that flubendazole inhibited cell viability and proliferation more efficiently in epi-MCF-7 cells than that in MCF-7 cells (Fig. S2B-C). Moreover, the percentage of CD44^high^/CD24^low^ population was dramatically reduced by 25% with flubendazole treatment in epi-MCF-7 cells (Fig. S2D). Taken together, these results indicated that flubendazole was preferably toxic to CS-like cells.

### Flubendazole induces differentiation and inhibits migration in breast cancer cells

**Figure 4:**
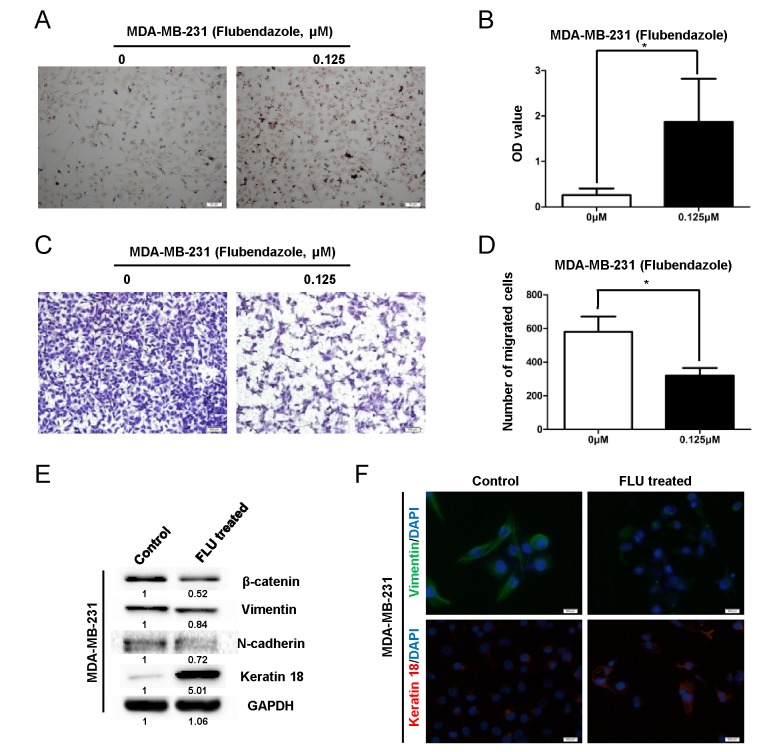
Flubendazole induces differentiation and inhibits migration in breast cancer cells MDA-MB-231 cells were treated with indicated concentration of flubendazole or vehicle control for 3 weeks. (A) The treated cells were subjected to Oil Red-O staining to identify lipid droplets. Images were taken under phase contrast microscopy (scale bar=50 μm). (B) Statistical results were presented as mean ± S.D. for three independent experiments (*p<0.05, Student's t test). (C) MDA-MB-231 cells with or without flubendazole treatment (0.125 μM, 3 weeks) were subjected to transwell assay as described in the methods. The representative images from three independent experiments were displayed (scale bar=500 μm). (D) The data were expressed as mean ± S.D. for experiments performed three times (*p<0.05, Student's t test) compared to control groups. (E) The expression of β-catenin, N-cadherin, Keratin 18 and Vimentin in MDA-MB-231 cells was identified by western blot assay before and after flubendazole treatment (0.125 μM, 3 weeks). (F) Expression of Vimentin and Keratin 18 was analyzed by IF analysis (scale bar=500 μm).

To explore whether flubendazole induces breast cancer cell differentiation, we performed Oil Red O staining in CS-like cell enriched MDA-MB-231 cells before and after flubendazole treatment (0.125 μM, 3 weeks) [31]. We observed that flubendazole dramatically increased positively staining cells (*p*<0.05) (Fig. 4A-B). Flubendazole treated MDA-MB-231 cells exhibited the downregulation of Vimentin and upregulation of Keratin 18 (Fig. 4E-F), indicating that flubendazole induced differentiation of breast cancer cells. Consistently, the percentage of CD44^high^/CD24^low^ population in MDA-MB-231 cells was reduced by 20% after treatment with flubendazole (Fig. S3A-B). These findings suggested that flubendazole might reduce CS-like cells through inducing cellular differentiation of breast cancer cells.

We then performed transwell assay to investigate the migration ability of those treated (0.125 μM, 3 weeks) or untreated MDA-MB-231 cells. Cell migration ability was crippled significantly by flubendazole from control group (580 ± 91.65 counts) to treated group (320 ± 45.82 counts, *p*=0.023) (Fig. 4C-D). Western blot assay showed that mesenchymal markers including β-catenin, N-cadherin and Vimentin were reduced by flubendazole (Fig. 4E). In contrast, epithelial marker Keratin 18 was elevated remarkably (Fig. 4E). Moreover, flubendazole reduced Vimentin and increased Keratin 18 expression showed by immunofluorescence (IF) assay (Fig. 4F). These results demonstrated that flubendazole inhibited cell migration and reversed EMT phenotype in MDA-MB-231 cells.

### Flubendazole arrests cell cycle at G2/M phase and induces monopolar spindle formation through inhibiting tubulin polymerization in breast cancer cells

**Figure 5:**
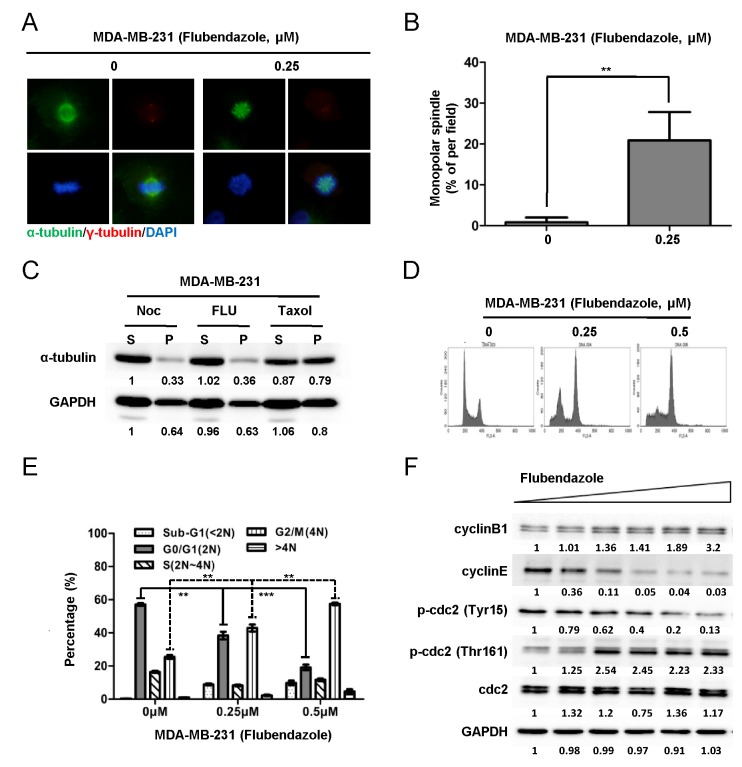
Flubendazole arrests cell cycle at G2/M phase and induces monopolar spindle formation through inhibiting tubulin polymerization in breast cancer cells (A) MDA-MB-231 cells were treated with vehicle (DMSO) and 0.25 μM flubendazole (dissolved in DMSO) for 24 hr, respectively. The expression of α-tubulin (green) and γ-tubulin (red) was analyzed by IF assay. Nuclear was stained with DAPI (blue). Morphological changes were observed under fluorescence microscopy (40×). (B) Graphs were statistically analyzed from five random fields (**p<0.01, Student's t test). (C) MDA-MB-231 cells were treated with nocodazole, flubendazole and taxol for 24 hr, respectively. Then, the cells were lysed and fractionated from cytosol (supernatant, S) to cytoskeletal (pellet, P) extracts. The extracts were subjected to western blot analysis for α-tubulin and GAPDH expression analysis. The quantification of blot density was shown in the figure. (D) MDA-MB-231 cells were treated with 0, 0.25, and 0.5 μM flubendazole for 24 hr, respectively. Cells were fixed and stained with PI. Cell cycle was analyzed on FACS Calibur flow cytometer. (E) The statistical results of three independent experiments were shown as mean ± S.D. (**p<0.01, ***p<0.001, Student's t test). (F) MDA-MB-231 cells were treated flubendazole in increasing doses (from 0 μM to 2 μM) for 24 hr and subjected to western blot to analyze cyclinB1, cyclinE, p-cdc2 (Tyr15), p-cdc2 (Thr161) and cdc2 expression.

To determine whether flubendazole inhibits tubulin polymerization, the spindle morphology was examined by IF assay. After 24 hr of treatment with 0.25 μΜ flubendazole or DMSO as control, MDA-MB-231 cells were fixed and stained against indicated antibodies. Cell morphology was analyzed in (Fig. 5A). The percentage of monopolar spindle was significantly increased from 1.03 ± 1.42% to 20.9 ± 6.92% (*p*=0.0025) with flubendazole treatment (Fig. 5B). Consistent results were observed in BT-549 cells (Fig. S4A-B). We further examined the effect of flubendazole against tubulin polymerization. MDA-MB-231 cells were treated with nocodazole (destabilizer of microtubule), flubendazole and taxol (stabilizer of microtubule) for 24 hr, respectively. The cells then were lysed and fractionated from cytosolic (supernatant, S) to cytoskeletal (pellet, P) extracts. The extracts were subjected to western blot assay. As shown in Fig. 5C, flubendazole suppressed tubulin polymerization similar with nocodazole treatment, while taxol accelerated tubulin polymerization. Similar results were observed in BT-549 cells (Fig. S4C). Thus, flubendazole induced monopolar spindle formation through inhibiting tubulin polymerization.

We also studied whether flubendazole blocked cell cycle progression in breast cancer cells. MDA-MB-231 cells were treated with 0, 0.25 and 0.5 μΜ flubendazole for 24 hr and then subjected to cell cycle profile analysis by flow cytometer. The percentage of G0/G1 (2N) phase cells was decreased from 58.31 ± 1.46% to 34.98 ± 3.97% and 16.44 ± 2.85% with the increasing flubendazole concentration (from 0 to 0.5 μM), (Fig. 5D-E). In contrast, the percentage of cells with 4N DNA content was increased from 25.49 ± 1.91% to 42.93 ± 3.65% and 55.55 ± 3.99% (Fig. 5D-E). The same phenomenon was also observed in BT-549 cells (Fig. S4D-E). Moreover, cell cycle markers were identified by western blot. In response to flubendazole treatment, both MDA-MB-231 and BT-549 cells showed increased phosphorylation of cdc2 at threonine 161 and decreased phosphorylation at tyrosine 15. These results indicated continuous cdc2 activation resulted from mitotic arrest. The increased expression of cyclinB1 and decreased expression of cyclinE suggested a G2/M arrest by flubendazole treatment (Fig. 5F and Fig. S4F). Taken together, these data demonstrated that flubendazole arrested cell cycle at G2/M phase through inhibiting tubulin polymerization.

### Flubendazole enhances the cytotoxic activity of fluorouracil and doxorubicin in breast cancer cells

**Figure 6:**
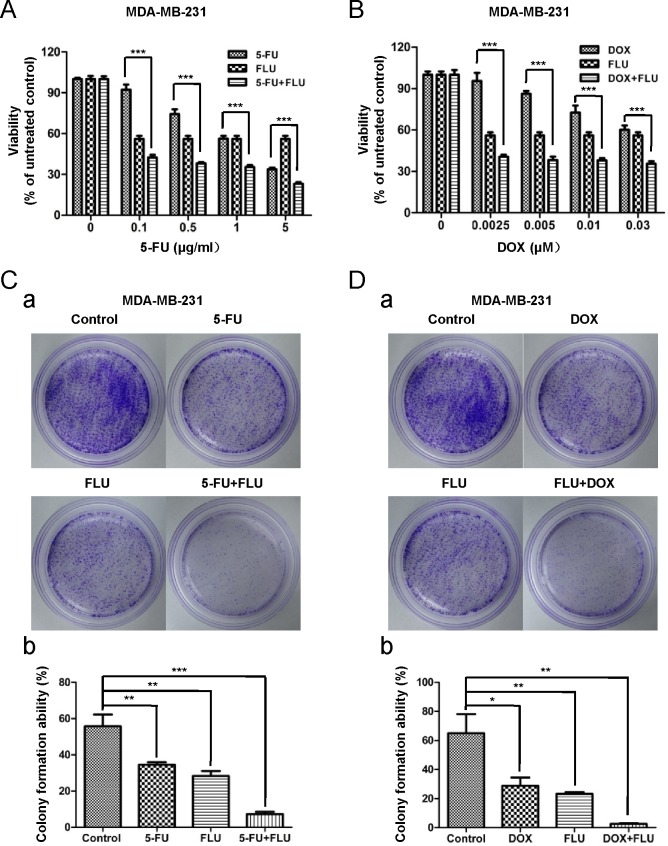
Flubendazole enhances the cytotoxic activity of fluorouracil and doxorubicin in breast cancer cells (A) MDA-MB-231 cells were treated with increasing doses of fluorouracil (5-FU, from 0 to 5 μg/ml), indicated dose of flubendazole and the combination of both. After 72 hr of incubation, cell viability was measured by MTT assay. (B) MDA-MB-231 cells were treated with increasing doses of doxorubicin (DOX, from 0 to 0.03 μM), indicated dose of flubendazole and the combination of both. After 72 hr of incubation, cell viability was measured by MTT assay. Data were presented as mean ± S.D. of three independent experiments (***p<0.001, Student's t test) (C) Dissociated MDA-MB-231 cells were seeded in 6-cm dishes and treated with indicated concentration of flubendazole, 5-FU, the combination of flubendazole and 5-FU and vehicle control for 9 days. Representative images of the colonies were recorded (a). Data were expressed as mean ± S.D. of three independent experiments (**p<0.01, ***p<0.001, Student's t test), (b). (D) Dissociated MDA-MB-231 cells were seeded in 6-cm dishes and treated with indicated concentration of flubendazole, DOX, the combination of flubendazole and DOX and vehicle control for 9 days. Representative images of the colonies were recorded (a). Data were presented as mean ± S.D. of three independent experiments (*p<0.05, **p<0.01, Student's t test), (b).

We further investigated whether flubendazole enhanced the cytotoxicity of conventional chemotherapy drugs such as fluorouracil and doxorubicin. MDA-MB-231 cells were treated with increasing concentration of fluorouracil (from 0 to 5 μg/ml) or doxorubicin (from 0 to 0.03 μM) with or without flubendazole. Cell viability was measured by MTT assay after 72 hr of incubation (Fig. 6A-B). Flubendazole treatment enhanced significantly the cytotoxic effect of fluorouracil and doxorubicin in breast cancer cells. The same results were observed in BT-549 cells (Fig. S5A-B).

To gain further evidence about the synergic effects of flubendazole and conventional chemotherapy drugs, colony formation assay was performed. As shown in Fig. 6C-D (a), fluorouracil (0.5 μg/ml), doxorubicin (0.01 μM) or flubendazole (0.125 μM) alone only partially reduced the colony formation. While conventional chemotherapy drugs (fluorouracil or doxorubicin) combined with flubendazole significantly inhibited the colony formation ability in MDA-MB-231 cells (Fig. 6C-D). Consistent results were shown in BT-549 cells (Fig. S5C-D). Taken together, flubendazole enhanced conventional chemotherapy drugs (fluorouracil or doxorubicin) efficiency, indicating flubendazole might function as a potential anti-cancer agent in combination therapy.

## DISCUSSION

Our data provided evidences for the application of flubendazole as anti-CS-like cells agent for breast cancer therapy by revealing several findings: (1) flubendazole inhibited breast cancer cell proliferation *in vitro* and suppressed tumor growth i*n vivo*; (2) flubendazole reduced breast CS-like cells evidenced by loss of CD44^high^/CD24^low^ population, reduction of mammospheres and the suppressed expression of stem cell related genes (*c-myc*, *oct4*, *sox2, nanog* and *cyclinD1*); (3) flubendazole induced cell differentiation, decreased cell migration and overcame drug resistance in breast cancer cells; (4) mechanistically, flubendazole induced monopolar spindle formation by inhibiting tubulin polymerization followed by cell cycle blockage at G2/M phase in breast cancer cells. These new findings implied that the conventional anthelmintic drug flubendazole could be applied as a novel anti-cancer agent for breast cancer therapy by targeting CS-like cells.

Recent studies report that the first-line drugs for treating other disease might be repurposed for targeting CS-like cells in breast cancer [32-35]. For instance, metformin, a hypoglycemic drug, reduced the incidence of various cancers in diabetic patients [32, 33]. Metformin suppressed tumor growth in xenografts by preferentially inhibiting nuclear translocation of NF-κB and phosphorylation of STAT3 in cancer stem cells [34]. All-trans retinoic acid, another first-line drug for leukemia, suppressed the mammosphere-forming ability which was correlated with the reduced expression of SOX2 and elevated expression of its antagonist CDX2 in breast CS-like cells [35].  The FDA approved flubendazole is extensively used as an efficacious anthelmintic drug in gastrointestinal parasites disease [21-23]. Here, we demonstrated that flubendazole significantly reduced CD44^high^/CD24^low^ population (Fig. 3A), sphere formation (Fig. 3C) and stem cell related genes (*c-myc*, *oct4*, *sox2, nanog* and *cyclinD1*) expression (Fig. 3I). Thus, flubendazole could be repurposed as a novel agent for targeting CS-like cells in breast cancer therapy.

Chemoresistance is a major obstacle to successful treatment of cancer. CSCs are considered to be resistant to chemotherapy due to their expressing drug pumps (ABC transporters) [36, 37]. Similarly, cancer stem-like cells could acquire drug transporters rendering cells chemoresistance [38, 39]. Therefore, strategies targeting breast CS-like cells might overcome chemotherapy resistance. For instance, Aurora-A inhibitor AKI603 combined with epirubicin significantly eliminates the tumor initiating cells and overcomes drug resistance in breast cancer [30]. Here, we demonstrated that flubendazole enhanced cytotoxic activity of fluorouracil and doxorubicin by targeting breast CS-like cells (Fig. 6). Strikingly, epirubicin resistant breast cancer cells that had an accumulation of CD44^high^/CD24^low^ population were sensitized to flubendazole (Fig. S2). Taken together, flubendazole could overcome chemoresistance and enhance the efficiency of conventional therapy. Thus, Flubendazole displayed potential advantages in clinical application for supplementing the shortage of conventional therapy.

Both cancer stem-like cell properties and EMT contribute to lung and liver metastasis in breast and colorectal cancer [40, 41]. Peptides (P17 and P144) targeting EMT also inhibit the expression of CD44 and SOX2 [41]. Keratin 18 is considered as a differentiation and epithelial marker, indicating differentiation and mesenchymal-epithelial transition (MET) of breast cancer cells [42, 43], while Vimentin functions as a dedifferentiation and mesenchymal marker in breast cancer cells [44, 45]. In the present study, we found that flubendazole treated MDA-MB-231 cells expressed high level of Keratin 18 and low level of β-catenin, N-cadherin and Vimentin (Fig. 4E-F), indicative of cell differentiation and EMT reversion. Meanwhile, the migration ability was crippled significantly by flubendazole (Fig. 4C-D). Therefore, flubendazole might be a hopeful candidate in breast cancer treatment.

The mechanism of flubendazole suppressing CS-like cells is not fully understood. Since CS-like cells are proliferating and actively cycling [14, 15], cell cycle blockage might affect the CS-like cell properties. Here, we reported that flubendazole arrested cell cycle at G2/M phase through interfering tubulin-microtubule equilibrium (Fig. 5 and Fig. S4). Mechanistically, these data suggested that breast CS-like cell capability was suppressed by the blockage of cell cycle progression. Thus, these new findings open an avenue for targeting CS-like cells through blocking cell cycle progression.

Flubendazole has been extensively studied for antihelmintic use in animal and humans with favorable toxicology profiles [22, 25, 46]. Consistently, our data displayed that flubendazole treatment (25 mg/kg, daily) did not alter gaining weight of nude mice during the experiment compared with the control group (Fig. 2D), indicating low toxicity to the mice. However, poor absorption is still a challenge for clinical application of flubendazole [29]. Flubendazole produces low plasma concentrations in hosts due to its high lipophilicity [22, 24]. Recent studies report that solid dispersion technique and emulsion cross-linking volatile technique are utilized to enhance the bioavalibility of albendazole that has the similar absorption properties and structure with flubendazole [47-49]. These methods for improving bioavalibility shed lights on the application of flubendazole in the clinical test.

In summary, we demonstrated that flubendazole, the FDA approved drug, exhibited a potential role in suppressing CS-like cells, inducing cell differentiation and reducing cell migration in poorly differentiated breast cancer cells. We also revealed a mechanism that flubendazole targeted breast CS-like cells through blocking cell cycle progression. Considering the safety profile and efficacy of flubendazole, our preclinical data rendered flubendazole as a viable therapeutic strategy for the treatment of breast cancer.

## MATERIALS AND METHODS

### Reagents

Flubendazole was offered by Yang group at Dalian University of Technology. Taxol was purchased from Sigma. Fluorouracil was purchased from Shanghai Sangon Company. Doxorubicin was purchased from Beyotime Institute of Biotechnology. Flubendazole was prepared in DMSO. Others were prepared in filtered dH_2_O.

### Cell culture

The human breast cancer cell lines (MDA-MB-231, BT-549, SK-BR-3 and MCF-7) were purchased from American Type Culture Collection (ATCC). The epi-MCF-7 cell line was obtained from professor Lam (Imperial College London UK, London, United Kingdom) and was previously described [50]. MDA-MB-231, SK-BR-3, MCF-7 and epi-MCF-7 cell lines were maintained in Dulbecco`s modified Eagle`s medium (DMEM, Invitrogen Corp) supplemented with 10% fetal bovine serum (Invitrogen Corp). BT-549 cell line was maintained in RPMI-1640 (Invitrogen Corp) supplemented with 10% fetal bovine serum (Invitrogen Corp). All cells were incubated at 37 °C in a humidified incubator containing 5% CO_2_.

### MTT assay and proliferation assay

MDA-MB-231, BT-549, SK-BR-3 and MCF-7 cells were seeded in 96-well microplates and cultured with various doses of flubendazole for 24, 48 and 72 hr, respectively. MTT solution (5 mg/ml, Sigma Aldrich) was added to cells and incubated for another 3 hr at 37 °C. The optical densities (OD) were measured at absorbance 570 nm. Cell proliferation was performed by cell counting assay. We seeded the same amount of cells into 24-well plate and counted for 3 continuous days monitoring the cell growth.

### Tumor growth in xenografts

MDA-MB-231 cells (2×10^6^) were inoculated into the right flank of 4~6 weeks old female nude mice (n=6), all of which developed tumors in 7 days with size of ~100 mm^3^. The mice were randomly distributed into two groups to receive 25 mg/kg flubendazole in 0.9% NaCl and 0.01% Tween-80 or vehicle control (0.9% NaCl and 0.01% Tween-80) once daily by intraperitoneal injection. Tumor volume (a×b^2^/2; a represented the greatest diameter, b was the diameter perpendicular to a) was measured by calipers twice every week. Other indicators of general health such as body weight, feeding behavior and motor activity of each animal were also monitored. After administration of flubendazole or vehicle for 16 days, the mice were euthanized and the tumor xenografts were immediately dissected, weighted, stored and fixed. Investigation has been conducted in accordance with the ethical standards and according to the Declaration of Helsinki and according to national and international guidelines and has been approved by the institutional animal care and use committee of Dalian Medical University.

### Flow cytometry analysis

Cells exposed to indicated concentration of flubendazole for 72 hr, were trypsinized, washed twice with PBS by centrifugation (1,000 rpm, 5 min), and blocked (0.5% BSA/PBS) for 10 min. Collected cells were then incubated with anti-CD24 PE (eBioscience) and anti-CD44 FITC (eBioscience) or isotype controls antibodies according to manufacturer's instructions for 30 min at 4 °C protected from light. Then, cells were suspended in 1 ml PBS and subjected to flow cytometry analysis (Beckman). Side-scatter and forward-scatter profiles were used to eliminate cell doublets [51]. The statistical results of three independent experiments were presented.

### Mammosphere formation assay

Dissociated single cells (1,000) were seeded in 6-well ultra-low attachment plates (Corning Costar). Cells were cultured with serum-free DMEM/F-12 (Invitrogen Corp) supplemented with 20 ng/ml epithelial growth factor (EGF, Sigma Aldrich), 20 ng/ml basic fibroblast growth factor (bFGF, PeproTech) and B27 in the absence and presence of flubendazole for 9 days. All spheres were photographed by invert microscope (10×, Olympus). The number and diameter of spheres were calculated by Image pro plus 6.0 (Olympus).

### Oil-Red-O staining

MDA-MB-231 cells were treated with 0.125 μΜ flubendazole or vehicle control for 3 weeks. Oil-Red-O staining for the detection of lipid droplets was performed as previously described [31]. Images were obtained under microscope.

### Migration assay

For the migration assay, 5,000 cells with or without indicated concentration of flubendazole were seeded on the matrigel coated Membrane in the upper chamber (24-well insert, 8 μm, Corning Costar). DMEM supplemented with 20% serum was used as an attractant in the lower chamber. After incubated for 24 hr, cells that migrated through the membrane were fixed with 70% ethanol and stained with 0.4% crystal violet (Shanghai Sangon Company). The stained cell images were captured by microscope, and five random fields at 10× magnification were counted. Results represented the average of triplicate samples from three independent experiments [51].

### Immunofluorescence analysis

Cells were grown on glass cover lips in the presence or absence of flubendazole for 24 hr. After fixed in 4% (v/v) formaldehyde/PBS, Cells were permeabilized (0.5% TritonX-100/PBS) and blocked for 1 hr in 5% BSA/PBS. IF was performed at room temperature using Vimentin (Merck Millipore, 1:200), Keratin 18 (Cell Signaling Technology, 1:100), α-tubulin (Santa Cruz, 1:400) and γ-tubulin (Sigma Aldrich, 1:800) antibodies. Alexa-conjugated anti-IgG antibodies (Invitrogen Corp, 1:200) were used for secondary detection. DAPI (Sigma Aldrich, 1 μg/ml) was used for nuclear staining. Images were acquired using an Olympus microscope.

### DNA content analysis

DNA content analysis was performed using propidium iodide (PI) staining. Briefly, cells were seeded in 6-cm dishes and treated with indicated concentration of flubendazole for 24 hr. Both adherent cells and floating cells were collected for analysis. The cells were fixed in 70% cold ethanol, stained with 1 mg/ml PI and analyzed by FACS Calibur flow cytometer (Becton Dickinson). Fluorescence profiles represent the DNA content of the PI stained cells.

### *In vivo* tubulin polymerization and microtubule disassembly assays

The separation of insoluble polymerized microtubules from soluble tubulin dimmers were performed as described previously [52]. In the study, cells were treated with flubendazole (0.25 μM), nocodazole (0.25 μM) and taxol (20 nM) for 24 hr, respectively. Then, the floating mitotic cells were harvested. Equal numbers of mitotic cells (3×10^6^) were lysed for 10 min at 4 °C in 30 μl lysis buffer containing 20 mM Tris-HCl (pH = 6.8), 1 mM MgCl_2_, 2 mM EGTA, 0.5% NP40, 2 mM PMSF and fresh cocktail. Proteins in the supernatants (containing soluble tubulin) were separated from pellets (containing insoluble tubulin) by centrifugation (15,000g, 10 min). The pellets were continuingly lysed in 30 μl of RIPA, and centrifuged at 15,000g for 10 min. Then, the supernatant (insoluble tubulin) were collected. Equal amounts of soluble and insoluble samples were subjected to western blot analysis. Primary antibodies for α-tubulin (Santa Cruz, 1:2000) and GAPDH (Kang Chen bio-tech, 1:5000) were used at manufactures recommended dilutions.

### Western blot analysis

After incubated with varying concentration of flubendazole and vehicle (DMSO) for 48 hr, cells were harvested and lysed in RIPA buffer. Protein concentration was determined by Bradford assay. Briefly, cell lysates (20 μg) were separated by SDS-PAGE, transferred onto nitrocellulose membrane (Merck Millipore). The membranes were blocked and exposed to cyclinB1 (Becton Dickinson, 1:2000), cyclinE (Becton Dickinson, 1:1000), p-cdc2 (Tyr15, Cell Signaling Technology, 1:2000), p-cdc2 (Thr161, Cell Signaling Technology, 1:2000), cdc2 (Cell Signaling Technology, 1:1000), c-MYC (Cell Signaling Technology, 1:1000), OCT4 (Santa Cruz, 1:200), SOX2 (Shanghai Sangon Company, 1:500), NANOG (Abcam, 1:2000), Keratin 18 (Cell Signaling Technology, 1:500), cyclinD1 (Becton Dickinson, 1:500), N-cadherin (Abcam, 1:1000), Vimentin (Merck Millipore, 1:2000) and β-catenin (Merck Millipore, 1:2000) antibodies, followed by incubation with appropriate secondary antibodies (Thermo Fisher Scientific, 1:5000). Proteins were visualized with the ECL system from Bio-Rad. The Western blots shown were representative of at least three independent experiments. GAPDH (Kang Chen bio-tech, 1:5000) was used as the loading control.

### Colony formation assay

Dissociated 1,000 cells were seeded in 6-cm dishes and treated with indicated concentration of flubendazole, fluorouracil (or doxorubicin), vehicle control or combined flubendazole with fluorouracil (or doxorubicin) for 9 days. At day 9, cells were fixed and stained with 0.4% crystal violet for 10 min at room temperature. The number of colonies was counted [53].

### Statistical considerations and calculations of flubendazole IC50

Data were presented as mean ± S.D. of three independent experiments using GraphPad Software (Prism5 program). Statistics were calculated by SPSS software (version 16.0). The statistical significance between groups was determined using the Student's t test. *p*≤0.05 was considered as statistically significant. IC50s were calculated using a nonlinear regression using one site competition curve [54].

## SUPPLEMENTARY MATERIAL, FIGURES



## Abbreviations

FLU: flubendazole
CS-like cell: cancer stemlike cell
CSC: cancer stem cell
ALDH1: aldehyde dehydrogenase1
EMT: epithelial-mesenchymal transition
5-FU: fluorouracil
DOX: doxorubicin
OD: optical densities
Noc: nocodazole
PI: propidium iodide
S: supernatant
P: pellet
IC50: 50% inhibitory concentration
epi-MCF-7: epirubicin-resistant MCF-7 cells
EGF: epithelial growth factor
bFGF: basic fibroblast growth factor
Hh: Hedgehog
ROS: reactive oxygen species
IF: Immunofluorescence

## References

[1] Jemal A, Bray F, Center MM, Ferlay J, Ward E, Forman D (2011). Global cancer statistics. CA Cancer J Clin.

[2] Kakarala M, Wicha MS (2008). Implications of the cancer stem-cell hypothesis for breast cancer prevention and therapy. J Clin Oncol.

[3] Gianni L, Baselga J, Eiermann W, Porta VG, Semiglazov V, Lluch A, Zambetti M, Sabadell D, Raab G, Cussac AL, Bozhok A, Martinez-Agullo A, Greco M, Byakhov M, Lopez JJ, Mansutti M (2009). Phase III trial evaluating the addition of paclitaxel to doxorubicin followed by cyclophosphamide, methotrexate, and fluorouracil, as adjuvant or primary systemic therapy: European Cooperative Trial in Operable Breast Cancer. J Clin Oncol.

[4] Peto R, Davies C, Godwin J, Gray R, Pan HC, Clarke M, Cutter D, Darby S, McGale P, Taylor C, Wang YC, Bergh J, Di Leo A, Albain K, Swain S, Piccart M (2012). Comparisons between different polychemotherapy regimens for early breast cancer: meta-analyses of long-term outcome among 100,000 women in 123 randomised trials. Lancet.

[5] Mani SA, Guo W, Liao MJ, Eaton EN, Ayyanan A, Zhou AY, Brooks M, Reinhard F, Zhang CC, Shipitsin M, Campbell LL, Polyak K, Brisken C, Yang J, Weinberg RA (2008). The epithelial-mesenchymal transition generates cells with properties of stem cells. Cell.

[6] Li X, Lewis MT, Huang J, Gutierrez C, Osborne CK, Wu MF, Hilsenbeck SG, Pavlick A, Zhang X, Chamness GC, Wong H, Rosen J, Chang JC (2008). Intrinsic resistance of tumorigenic breast cancer cells to chemotherapy. J Natl Cancer Inst.

[7] Phillips TM, McBride WH, Pajonk F (2006). The response of CD24(−/low)/CD44+ breast cancer-initiating cells to radiation. J Natl Cancer Inst.

[8] Ginestier C, Hur MH, Charafe-Jauffret E, Monville F, Dutcher J, Brown M, Jacquemier J, Viens P, Kleer CG, Liu S, Schott A, Hayes D, Birnbaum D, Wicha MS, Dontu G (2007). ALDH1 is a marker of normal and malignant human mammary stem cells and a predictor of poor clinical outcome. Cell Stem Cell.

[9] Al-Hajj M, Wicha MS, Benito-Hernandez A, Morrison SJ, Clarke MF (2003). Prospective identification of tumorigenic breast cancer cells. Proc Natl Acad Sci U S A.

[10] Nicolini A, Ferrari P, Fini M, Borsari V, Fallahi P, Antonelli A, Carpi A, Miccoli P (2011). Cancer stem cells: perspectives of new therapeutical approaches for breast cancer. Front Biosci (Schol Ed).

[11] Morel AP, Lievre M, Thomas C, Hinkal G, Ansieau S, Puisieux A (2008). Generation of breast cancer stem cells through epithelial-mesenchymal transition. PLoS One.

[12] Bane A, Viloria-Petit A, Pinnaduwage D, Mulligan AM, O'Malley FP, Andrulis IL (2013). Clinical-pathologic significance of cancer stem cell marker expression in familial breast cancers. Breast Cancer Res Treat.

[13] Yanamoto S, Yamada S, Takahashi H, Naruse T, Matsushita Y, Ikeda H, Shiraishi T, Seki S, Fujita S, Ikeda T, Asahina I, Umeda M (2014). Expression of the cancer stem cell markers CD44v6 and ABCG2 in tongue cancer: effect of neoadjuvant chemotherapy on local recurrence. Int J Oncol.

[14] Blagosklonny MV (2006). Target for cancer therapy: proliferating cells or stem cells. Leukemia.

[15] Blagosklonny MV (2007). Cancer stem cell and cancer stemloids: from biology to therapy. Cancer Biol Ther.

[16] Zhang XY, Varthi M, Sykes SM, Phillips C, Warzecha C, Zhu W, Wyce A, Thorne AW, Berger SL, McMahon SB (2008). The putative cancer stem cell marker USP22 is a subunit of the human SAGA complex required for activated transcription and cell-cycle progression. Mol Cell.

[17] Tarasov V, Jung P, Verdoodt B, Lodygin D, Epanchintsev A, Menssen A, Meister G, Hermeking H (2007). Differential regulation of microRNAs by p53 revealed by massively parallel sequencing: miR-34a is a p53 target that induces apoptosis and G1-arrest. Cell Cycle.

[18] Liu C, Kelnar K, Liu B, Chen X, Calhoun-Davis T, Li H, Patrawala L, Yan H, Jeter C, Honorio S, Wiggins JF, Bader AG, Fagin R, Brown D, Tang DG (2011). The microRNA miR-34a inhibits prostate cancer stem cells and metastasis by directly repressing CD44. Nat Med.

[19] Lee DF, Su J, Ang YS, Carvajal-Vergara X, Mulero-Navarro S, Pereira CF, Gingold J, Wang HL, Zhao R, Sevilla A, Darr H, Williamson AJ, Chang B, Niu X, Aguilo F, Flores ER (2012). Regulation of embryonic and induced pluripotency by aurora kinase-p53 signaling. Cell Stem Cell.

[20] Yan M, Liu QQ (2013). Targeted therapy: tailoring cancer treatment. Chin J Cancer.

[21] Ceballos L, Elissondo C, Sanchez Bruni S, Denegri G, Lanusse C, Alvarez L (2011). Comparative performances of flubendazole and albendazole in cystic echinococcosis: ex vivo activity, plasma/cyst disposition, and efficacy in infected mice. Antimicrob Agents Chemother.

[22] Ceballos L, Virkel G, Elissondo C, Canton C, Canevari J, Murno G, Denegri G, Lanusse C, Alvarez L (2013). A pharmacology-based comparison of the activity of albendazole and flubendazole against Echinococcus granulosus metacestode in sheep. Acta Trop.

[23] Ceballos L, Elissondo M, Bruni SS, Denegri G, Alvarez L, Lanusse C (2009). Flubendazole in cystic echinococcosis therapy: pharmaco-parasitological evaluation in mice. Parasitol Int.

[24] Mackenzie CD, Geary TG (2011). Flubendazole: a candidate macrofilaricide for lymphatic filariasis and onchocerciasis field programs. Expert Rev Anti Infect Ther.

[25] Dominguez-Vazquez A, Taylor HR, Greene BM, Ruvalcaba-Macias AM, Rivas-Alcala AR, Murphy RP, Beltran-Hernandez F (1983). Comparison of flubendazole and diethylcarbamazine in treatment of onchocerciasis. Lancet.

[26] Katiyar SK, Gordon VR, McLaughlin GL, Edlind TD (1994). Antiprotozoal activities of benzimidazoles and correlations with beta-tubulin sequence. Antimicrob Agents Chemother.

[27] Lacey E (1988). The role of the cytoskeletal protein, tubulin, in the mode of action and mechanism of drug resistance to benzimidazoles. Int J Parasitol.

[28] Kralova V, Hanusova V, Stankova P, Knoppova K, Canova K, Skalova L (2013). Antiproliferative effect of benzimidazole anthelmintics albendazole, ricobendazole, and flubendazole in intestinal cancer cell lines. Anticancer Drugs.

[29] Spagnuolo PA, Hu J, Hurren R, Wang X, Gronda M, Sukhai MA, Di Meo A, Boss J, Ashali I, Beheshti Zavareh R, Fine N, Simpson CD, Sharmeen S, Rottapel R, Schimmer AD (2010). The antihelmintic flubendazole inhibits microtubule function through a mechanism distinct from Vinca alkaloids and displays preclinical activity in leukemia and myeloma. Blood.

[30] Zheng FM, Long ZJ, Hou ZJ, Luo Y, Xu LZ, Xia JL, Lai XJ, Liu JW, Wang X, Kamran M, Yan M, Shao SJ, Lam EW, Wang SW, Lu G, Liu Q (2014). A novel small molecule aurora kinase inhibitor attenuates breast tumor-initiating cells and overcomes drug resistance. Mol Cancer Ther.

[31] Caldon CE, Lee CS, Sutherland RL, Musgrove EA (2008). Wilms' tumor protein 1: an early target of progestin regulation in T-47D breast cancer cells that modulates proliferation and differentiation. Oncogene.

[32] Evans JM, Donnelly LA, Emslie-Smith AM, Alessi DR, Morris AD (2005). Metformin and reduced risk of cancer in diabetic patients. Br Med J.

[33] Jiralerspong S, Palla SL, Giordano SH, Meric-Bernstam F, Liedtke C, Barnett CM, Hsu L, Hung MC, Hortobagyi GN, Gonzalez-Angulo AM (2009). Metformin and pathologic complete responses to neoadjuvant chemotherapy in diabetic patients with breast cancer. J Clin Oncol.

[34] Hirsch HA, Iliopoulos D, Struhl K (2013). Metformin inhibits the inflammatory response associated with cellular transformation and cancer stem cell growth. Proc Natl Acad Sci U S A.

[35] Bhat-Nakshatri P, Goswami CP, Badve S, Sledge GW (2013). and Nakshatri H. Identification of FDA-approved drugs targeting breast cancer stem cells along with biomarkers of sensitivity. Sci Rep.

[36] Chaudhary PM, Roninson IB (1991). Expression and activity of P-glycoprotein, a multidrug efflux pump, in human hematopoietic stem cells. Cell.

[37] Kim M, Turnquist H, Jackson J, Sgagias M, Yan Y, Gong M, Dean M, Sharp JG, Cowan K (2002). The multidrug resistance transporter ABCG2 (breast cancer resistance protein 1) effluxes Hoechst 33342 and is overexpressed in hematopoietic stem cells. Clin Cancer Res.

[38] Patrawala L, Calhoun T, Schneider-Broussard R, Zhou J, Claypool K, Tang DG (2005). Side population is enriched in tumorigenic, stem-like cancer cells, whereas ABCG2+ and ABCG2- cancer cells are similarly tumorigenic. Cancer Res.

[39] Hirschmann-Jax C, Foster AE, Wulf GG, Nuchtern JG, Jax TW, Gobel U, Goodell MA, Brenner MK (2004). A distinct “side population” of cells with high drug efflux capacity in human tumor cells. Proc Natl Acad Sci U S A.

[40] Hayashida T, Jinno H, Kitagawa Y, Kitajima M (2011). Cooperation of cancer stem cell properties and epithelial-mesenchymal transition in the establishment of breast cancer metastasis. J Oncol.

[41] Zubeldia IG, Bleau AM, Redrado M, Serrano D, Agliano A, Gil-Puig C, Vidal-Vanaclocha F, Lecanda J, Calvo A (2013). Epithelial to mesenchymal transition and cancer stem cell phenotypes leading to liver metastasis are abrogated by the novel TGFbeta1-targeting peptides P17 and P144. Exp Cell Res.

[42] Papi A, Guarnieri T, Storci G, Santini D, Ceccarelli C, Taffurelli M, De Carolis S, Avenia N, Sanguinetti A, Sidoni A, Orlandi M, Bonafe M (2012). Nuclear receptors agonists exert opposing effects on the inflammation dependent survival of breast cancer stem cells. Cell Death Differ.

[43] Ventayol M, Vinas JL, Sola A, Jung M, Brune B, Pi F, Mastora C, Hotter G (2014). miRNA let-7e targeting MMP9 is involved in adipose-derived stem cell differentiation toward epithelia. Cell Death Dis.

[44] Sommers CL, Byers SW, Thompson EW, Torri JA, Gelmann EP (1994). Differentiation state and invasiveness of human breast cancer cell lines. Breast Cancer Res Treat.

[45] Paccione RJ, Miyazaki H, Patel V, Waseem A, Gutkind JS, Zehner ZE, Yeudall WA (2008). Keratin down-regulation in vimentin-positive cancer cells is reversible by vimentin RNA interference, which inhibits growth and motility. Mol Cancer Ther.

[46] Squires S, Fisher M, Gladstone O, Rogerson S, Martin P, Martin S, Lester H, Sygall R, Underwood N (2012). Comparative efficacy of flubendazole and a commercially available herbal wormer against natural infections of Ascaridia galli, Heterakis gallinarum and intestinal Capillaria spp. in chickens. Vet Parasitol.

[47] Kohri N, Yamayoshi Y, Xin H, Iseki K, Sato N, Todo S, Miyazaki K (1999). Improving the oral bioavailability of albendazole in rabbits by the solid dispersion technique. J Pharm Pharmacol.

[48] Liu Y, Wang XQ, Ren WX, Chen YL, Yu Y, Zhang JK, Bawudong D, Gu JP, Xu XD, Zhang XN (2013). Novel albendazole-chitosan nanoparticles for intestinal absorption enhancement and hepatic targeting improvement in rats. J Biomed Mater Res B Appl Biomater.

[49] Castro N, Marquez-Caraveo C, Brundage RC, Gonzalez-Esquivel D, Suarez AM, Gongora F, Jara A, Urizar J, Lanao JM, Jung H (2009). Population pharmacokinetics of albendazole in patients with neurocysticercosis. Int J Clin Pharmacol Ther.

[50] Millour J, de Olano N, Horimoto Y, Monteiro LJ, Langer JK, Aligue R, Hajji N, Lam EW (2011). ATM and p53 regulate FOXM1 expression via E2F in breast cancer epirubicin treatment and resistance. Mol Cancer Ther.

[51] Lu Y, Lu J, Li X, Zhu H, Fan X, Zhu S, Wang Y, Guo Q, Wang L, Huang Y, Zhu M, Wang Z (2014). MiR-200a inhibits epithelial-mesenchymal transition of pancreatic cancer stem cell. BMC Cancer.

[52] Wu YC, Yen WY, Ho HY, Su TL, Yih LH (2010). Glyfoline induces mitotic catastrophe and apoptosis in cancer cells. Int J Cancer.

[53] Digirolamo CM, Stokes D, Colter D, Phinney DG, Class R, Prockop DJ (1999). Propagation and senescence of human marrow stromal cells in culture: a simple colony-forming assay identifies samples with the greatest potential to propagate and differentiate. Br J Haematol.

[54] Ding WQ, Liu B, Vaught JL, Palmiter RD, Lind SE (2006). Clioquinol and docosahexaenoic acid act synergistically to kill tumor cells. Mol Cancer Ther.

