# Supplementary material for: Flubendazole, FDA-approved anthelmintic, targets breast cancer stem-like cells
Source: Oncotarget. 2015 Jan 21;6(8):6326–40. doi: 10.18632/oncotarget.3436 (PMC4467440; doi:10.18632/oncotarget.3436)
Supplement: Supplementary file 1 [file oncotarget-06-6326-s001.pdf]

Flubendazole, FDA-approved anthelmintic, targets breast cancer stem-like cells

Supplementary Material

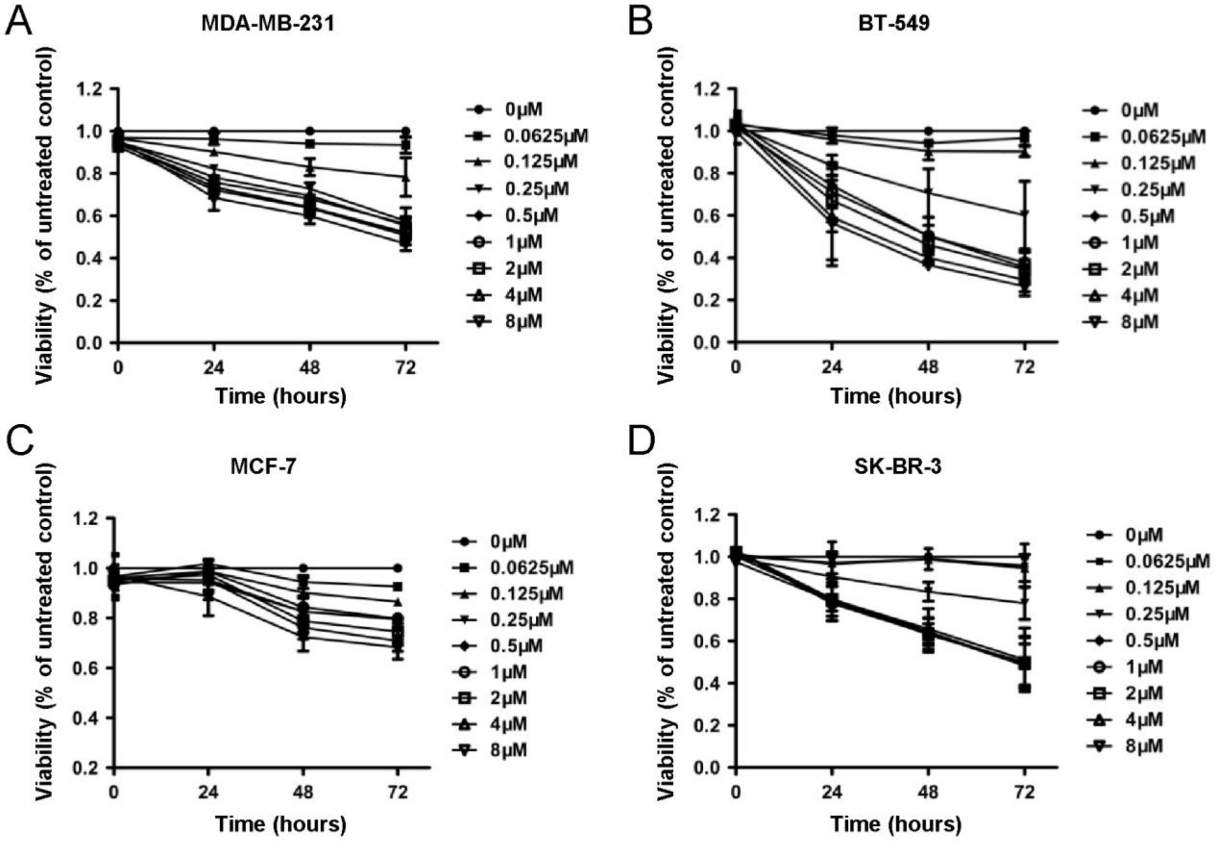

Supplemental figure 1: Flubendazole inhibits cell viability in breast cancer cells. (A) MDA-MB-231, (B) BT-549, (C) SK-BR-3 and (D) MCF-7 cells were treated with increasing doses of flubendazole, respectively. After 24, 48 and 72 hr of incubation, cell viability was measured by MTT assay. Data from three independent experiments were shown as mean ± S.D. compared with vehicle-treated breast cancer cells.

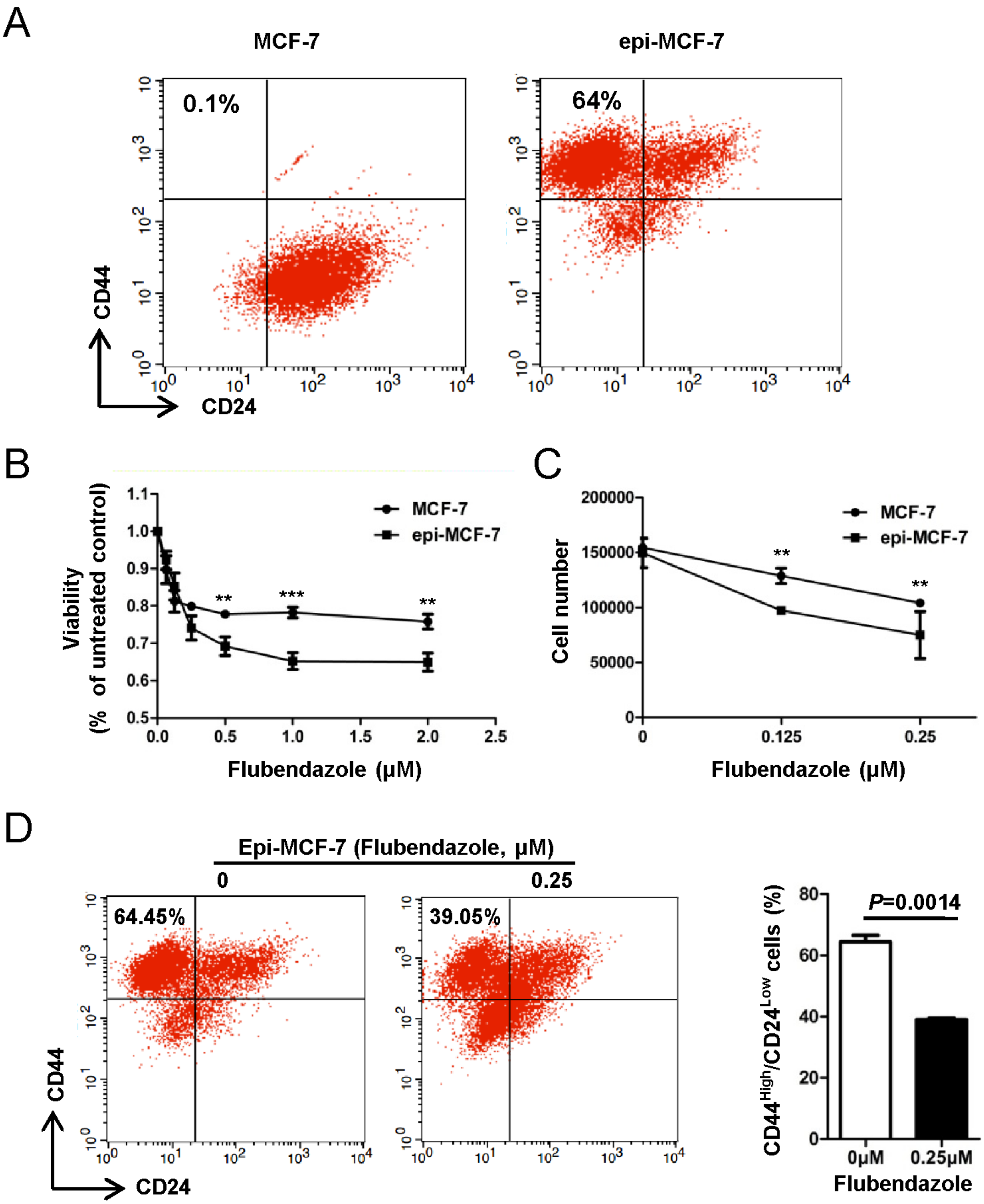

Supplemental figure 2: Flubendazole was preferably cytotoxic to CD44^{high}/CD24^{low} population. (A) CD44^{high}/CD24^{low} population was measured by flow cytometry analysis in MCF-7 and epi-MCF-7 cells. Representative results of CD44^{high}/CD24^{low} proportion in MCF-7 and epi-MCF-7 cells were presented. (B) MCF-7 and epi-MCF-7 cells were treated with

increasing doses of flubendazole (from 0.0625 μM to 2 μM) for 72 hr and then were subjected to MTT assay. Data were demonstrated as mean ± S.D. from three independent experiments (**p<0.01, ***p<0.001, Student's t test). (C) Both MCF-7 and epi-MCF-7 cells were treated with indicated doses of flubendazole (0, 0.125 and 0.25 μM), respectively. After incubating for 72 hr, cell counting assay was performed. Data were demonstrated as mean ± S.D. from three independent experiments (**p<0.01, Student's t test). (D) Epi-MCF-7 cells were treated with indicated doses of flubendazole for 48 hr. $CD44^{high}/CD24^{low}$ population was analyzed by flow cytometry analysis. The statistical results of $CD44^{high}/CD24^{low}$ proportion were presented. Data were demonstrated as mean ± S.D. (**p<0.01, Student's t test).

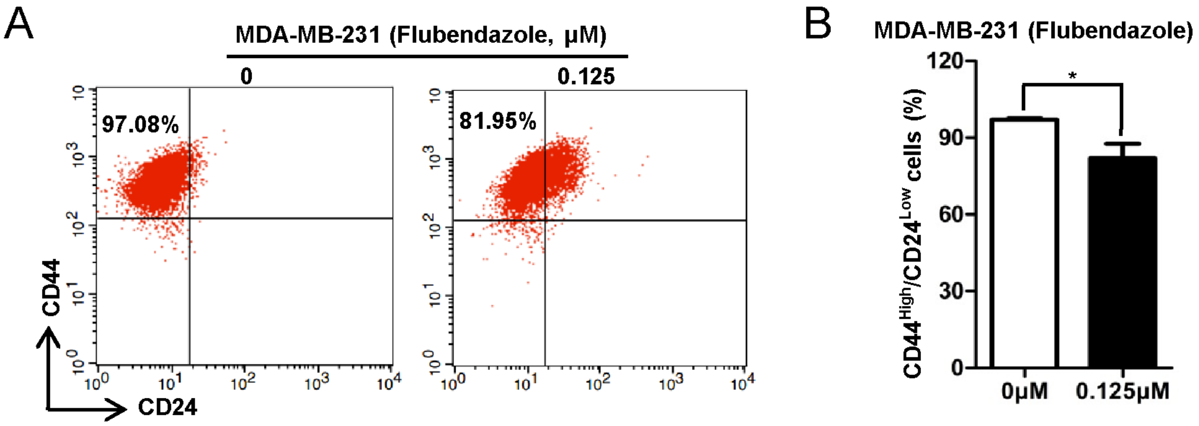

Supplemental figure 3: Breast CS-like cells were reduced by long term flubendazole treatment. (A) $CD44^{high}/CD24^{low}$ population was analyzed by flow cytometry analysis in flubendazole treated or untreated MDA-MB-231 cells. (B) The statistical results of three independent experiments were presented. Data were demonstrated as mean ± S.D. (*p<0.05, Student's t test).

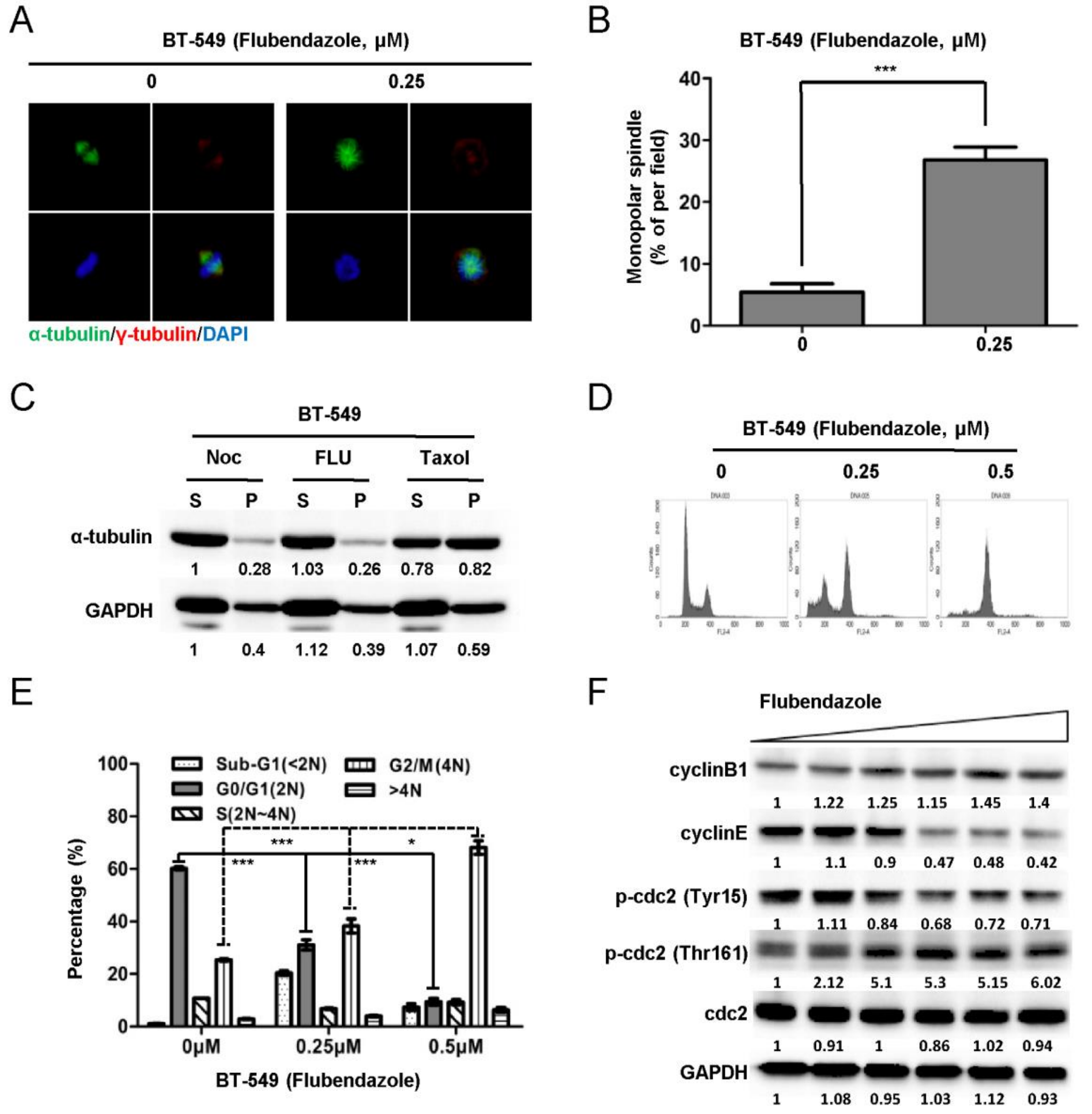

Supplemental figure 4: Flubendazole arrests cell cycle at G2/M phase and induces monopolar spindle formation by inhibiting tubulin polymerization in BT-549 cells. (A) BT-549 cells were treated with vehicle (DMSO) and 0.25 μM flubendazole (dissolved in DMSO) for 24 hr. Immunofluorescence staining was performed to analyze the expression of α-Tubulin (green) and γ-Tubulin (red). Nuclear was stained with DAPI (blue). Morphological changes were observed under fluorescence microscopy (40×). (B) Graphs were statistically analyzed from five random fields (****p*<0.001, Student's t test). (C) BT-549 cells were treated with nocodazole,

flubendazole and taxol for 24 hr, respectively. Then those cells were lysed and fractionated from cytosol (soluble, S) to cytoskeletal (pellet, P) extracts. The extracts were subjected to western blot to analyze α-tubulin expression, GAPDH acted as loading control. (D) BT-549 cells were treated with 0, 0.25, and 0.5 μM flubendazole for 24 hr, respectively. Cell cycle profile was analyzed on FACS Calibur flow cytometer. (E) The statistical results of three independent experiments were shown as mean ± S.D. (*p<0.05, ***p<0.001, Student's t test). (F) BT-549 cells were treated with increasing doses of flubendazole (from 0 μM to 2 μM) for 24 hr and subjected to western blot to analyze cyclinB1, cyclinE, p-cdc2 (Tyr15), p-cdc2 (Thr161) and cdc2 expression.

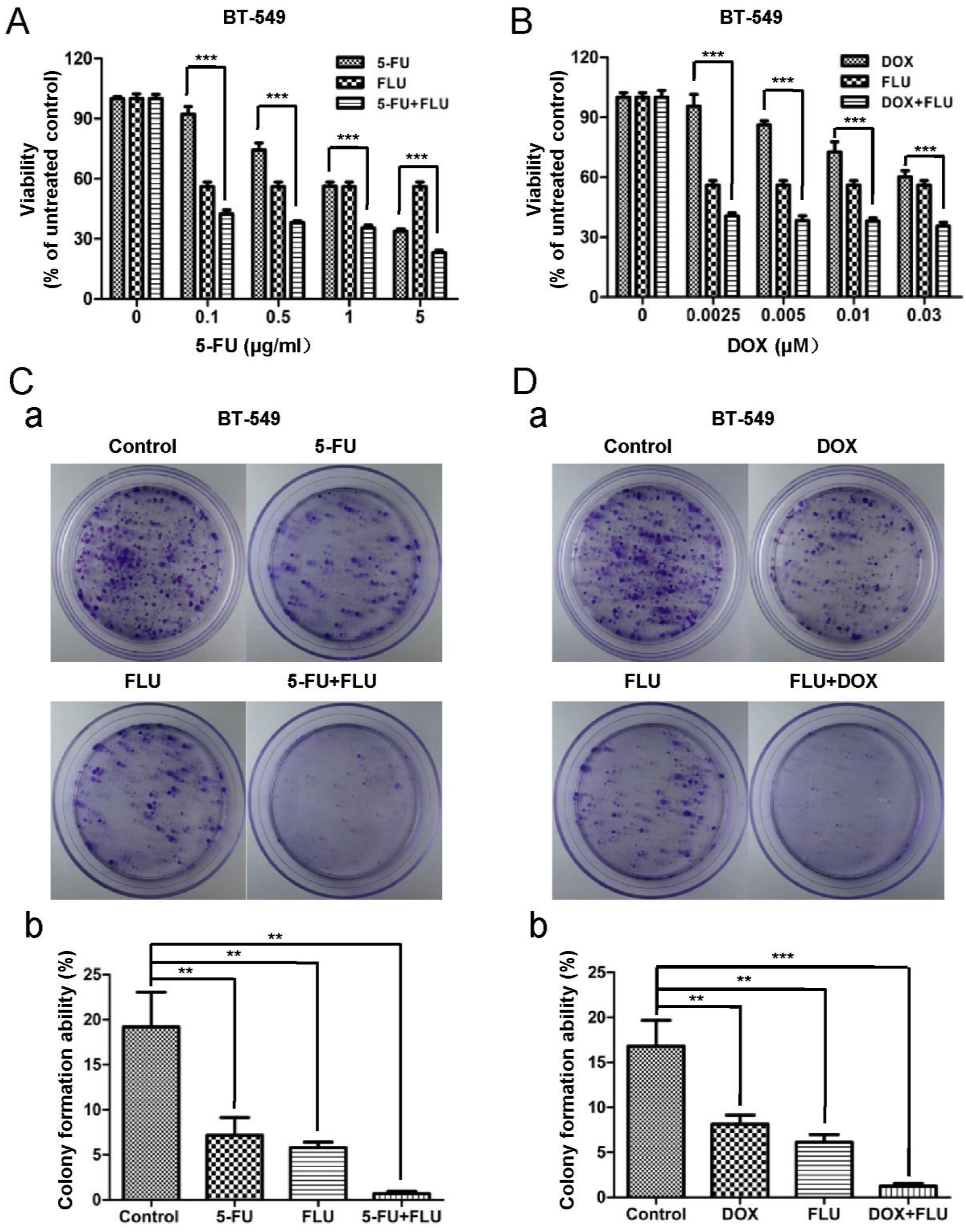

Supplemental Figure 5: Flubendazole enhances the cytotoxic activity of fluorouracil and doxorubicin in BT-549 cells. (A) BT-549 cells were treated with increasing doses of fluorouracil (5-FU), indicated doses of flubendazole and the combination of both. After 72 hr of

incubation, cell viability was measured by MTT assay. (B) BT-549 cells were treated with increasing concentrations of doxorubicin (DOX), indicated doses of flubendazole and the combination of both. After 72 hr of incubation, cell viability was measured by MTT assay. Data were presented as mean ± S.D. of three independent experiments (***$p<0.001$, Student's t test) (C) Dissociated BT-549 cells were seeded in 6-cm dishes and treated with indicated doses of flubendazole, 5-FU, the combination of flubendazole and 5-FU or vehicle control for 9 days. Representative images of the colonies were recorded (a). Data were expressed as mean ± S.D. of three independent experiments (**$p<0.01$, Student's t test), (b) (D) Dissociated BT-549 cells were seeded in 6-cm dishes and treated with indicated concentrations of flubendazole, DOX, the combination of flubendazole and DOX or vehicle control for 9 days. Representative images of the colonies were recorded (a). Data were expressed as mean ± S.D. of three independent experiments (**$p<0.01$, ***$p<0.001$, Student's t test), (b).
